# Supplementary material for: Antidepressant Screening Demonstrated Non-Monotonic Responses to Amitriptyline, Amoxapine and Sertraline in Locomotor Activity Assay in Larval Zebrafish
Source: Cells. 2021 Mar 26;10(4):738. doi: 10.3390/cells10040738 (PMC8066259; doi:10.3390/cells10040738)
Supplement: Supplementary file 1 [file cells-10-00738-s001.pdf]

Supplementary Data

Table S1. The P value of two-way ANOVA from each zebrafish larvae locomotor activity endpoints displayed in Figure 2 (* P < 0.05, ** P<0.01, *** P < 0.001, **** P < 0.0001). Abbreviation: Amitriptyline hydrochloride (AMY), amoxapine (AMO), atomoxetine hydrochloride (ATM), bupropion hydrochloride (BUP), doxepin hydrochloride (DOX) duloxetine hydrochloride (DLX), escitalopram oxalate (ESC), fluoxetine hydrochloride (FLX), imipramine hydrochloride (IMP), mianserin hydrochloride (MIA), milnacipran (MCP), moclobemide (MEM), and venlafaxine hydrochloride (VEN), mirtazapine (MRT), Paroxetine (PAR), Selegiline hydrochloride (SEG), Sertraline hydrochloride (SRT), Trazodone hydrochloride (TRA).

Behavior Endpoints	Antidepressants	Difference Between Means ± Standard Error (Treated – Control)	F(1, 190) (F (DFn, DFd))	P Value	Significance
Total Distance	MEM	-15.69 ± 4.476	12.28	0.0006	***
	TRA	-12.79 ± 4.552	7.889	0.0055	**
	FLX	-12.15 ± 4.374	7.717	0.0060	**
	SRT	-7.257 ± 4.967	2.135	0.1456	n.s.
	ESC	-2.823 ± 5.434	0.2699	0.6040	n.s.
	DLX	-17.08 ± 4.391	15.13	0.0001	***
	ATM	-9.736 ± 5.755	2.863	0.0923	n.s.
	VEN	-18.16 ± 4.529	16.09	< 0.0001	****
	IMP	-12.92 ± 4.388	8.670	0.0036	**
	PAR	-9.025 ± 5.038	3.290	0.0748	n.s.
	MRT	14.69 ± 8.184	3.223	0.0742	n.s.
	AMY	-2.730 ± 4.375	0.3894	0.5334	n.s.
	DOX	-13.97 ± 6.985	4.002	0.0469	*
	AMO	2.302 ± 4.976	0.2141	0.6441	n.s.
	BUP	-17.08 ± 4.559	14.04	0.0002	***
	MIA	-7.842 ± 5.105	2.380	0.1261	n.s.
	MCP	-20.06 ± 4.423	20.58	< 0.0001	****
	SEG	-22.51 ± 4.350	26.78	< 0.0001	****
Burst Movement Count	MEM	-2.808 ± 0.7238	15.04	0.0001	***
	TRA	0.1247 ± 0.8241	0.02291	0.8799	n.s.
	FLX	-0.3625 ± 0.7596	0.2277	0.6337	n.s.
	SRT	-2.832 ± 0.7296	15.06	0.0001	***
	ESC	-2.569 ± 0.7112	13.05	0.0004	***
	DLX	-4.723 ± 0.6909	46.74	< 0.0001	****
	ATM	-4.415 ± 0.6905	40.88	< 0.0001	****
	VEN	-4.940 ± 0.6907	51.17	< 0.0001	****
	IMP	-4.588 ± 0.6952	43.56	< 0.0001	****
	PAR	-3.925 ± 0.7501	27.38	< 0.0001	****
	MRT	-0.5326 ± 0.7678	0.4811	0.4888	n.s.
	AMY	1.977 ± 0.7659	6.660	0.0106	*

	**DOX**	-5.536 ± 0.6841	65.50	< 0.0001	****
	**AMO**	8.995 ± 1.656	29.50	< 0.0001	****
	**BUP**	-4.082 ± 0.6977	34.22	< 0.0001	****
	**MIA**	8.254 ± 1.339	38.02	< 0.0001	****
	**MCP**	-1.207 ± 0.7579	2.538	0.1128	n.s.
	**SEG**	-3.066 ± 0.7251	17.89	< 0.0001	****
**Rotation Count**	**MEM**	-0.1876 ± 0.03508	28.60	< 0.0001	****
	**TRA**	-0.1751 ± 0.03537	24.51	< 0.0001	****
	**FLX**	-0.1462 ± 0.03644	16.09	< 0.0001	****
	**SRT**	0.02257 ± 0.04374	0.2663	0.6065	n.s.
	**ESC**	-0.3993 ± 0.03696	1.167	0.2813	n.s.
	**DLX**	-0.2618 ± 0.03357	60.82	< 0.0001	****
	**ATM**	-0.2280 ± 0.03428	44.21	< 0.0001	****
	**VEN**	-0.2576 ± 0.03375	58.28	< 0.0001	****
	**IMP**	-0.2563 ± 0.03368	57.94	< 0.0001	****
	**PAR**	-0.2181 ± 0.03705	34.64	< 0.0001	****
	**MRT**	0.06788 ± 0.04006	2.871	0.0918	n.s.
	**AMY**	-0.1727 ± 0.03386	26.03	< 0.0001	****
	**DOX**	-0.3048 ± 0.03317	84.40	< 0.0001	****
	**AMO**	-0.07196 ± 0.04266	2.846	0.0932	n.s.
	**BUP**	-0.2993 ± 0.03320	81.30	< 0.0001	****
	**MIA**	-0.06988 ± 0.04494	2.418	0.1216	n.s.
	**MCP**	-0.2566 ± 0.03358	58.40	< 0.0001	****
	**SEG**	-0.2756 ± 0.03335	68.28	< 0.0001	****

Table S2. The two-way ANOVA test result details from each zebrafish larvae locomotor activity endpoint after treated with 3 different antidepressants in comparison to the control group displayed in Figure S2 (* $P < 0.05$, **** $P < 0.0001$). Abbreviation: Amitriptyline hydrochloride (AMY), amoxapine (AMO), Sertraline hydrochloride (SRT).

**Behavior Endpoints**	**Antidepressants**		**F Time (Row Factor) (F (DFn, DFd))**	**F Column Factor (F (DFn, DFd))**	**P Value**	**Significance**
Total Distance	**AMY**	1 ppb	F (10.55, 4747) = 38.66 P < 0.0001	F (3, 450) = 1.681 P = 0.1704	0.0111	*
		10 ppb			< 0.0001	****
		100 ppb			< 0.0001	****
	**AMO**	1 ppb	F (10.17, 4588) = 68.90 P < 0.0001	F (3, 451) = 26.96 P < 0.0001	< 0.0001	****
		10 ppb			< 0.0001	****
		100 ppb			< 0.0001	****
	**SRT**	1 ppb	F (10.73, 4838) = 76.08 P < 0.0001	F (3, 451) = 5.140 P = 0.0017	< 0.0001	****
		10 ppb			< 0.0001	****
		100 ppb			< 0.0001	****
	**AMY**	1 ppb	F (10.29, 4630) = 203.2	F (3, 450) = 42.40	< 0.0001	****

Burst Movement Count		10 ppb	P < 0.0001	P < 0.0001	< 0.0001	****
		100 ppb			< 0.0001	****
	**AMO**	1 ppb	F (8.971, 4046) = 299.4 P < 0.0001	F (3, 451) = 126.9 P < 0.0001	< 0.0001	****
		10 ppb			< 0.0001	****
		100 ppb			< 0.0001	****
	**SRT**	1 ppb	F (4.931, 2224) = 235.1 P < 0.0001	F (3, 451) = 15.19 P < 0.0001	< 0.0001	****
		10 ppb			< 0.0001	****
		100 ppb			< 0.0001	****
Rotation Count	**AMY**	1 ppb	F (22.92, 10312) = 62.56 P < 0.0001	F (3, 450) = 8.053 P < 0.0001	< 0.0001	****
		10 ppb			< 0.0001	****
		100 ppb			< 0.0001	****
	**AMO**	1 ppb	F (22.43, 10118) = 102.3 P < 0.0001	F (3, 451) = 45.38 P < 0.0001	< 0.0001	****
		10 ppb			< 0.0001	****
		100 ppb			< 0.0001	****
	**SRT**	1 ppb	F (20.62, 9300) = 66.22 P < 0.0001	F (3, 451) = 11.54 P < 0.0001	< 0.0001	****
		10 ppb			< 0.0001	****
		100 ppb			< 0.0001	****

Table S3. The Kruskal-Wallis test result details from each zebrafish larvae locomotor activity endpoint after treated with 3 different antidepressants in comparison to the control group displayed in Figure 4 (n.s. (not significant) P ≥ 0.05, * P < 0.05, ** P<0.01, *** P < 0.001, **** P < 0.0001). Abbreviation: Amitriptyline hydrochloride (AMY), amoxapine (AMO), Sertraline hydrochloride (SRT).

**Behavior Endpoints**		**Antidepressants**		**P Value**	**Significance**
Total Distance	Light Cycle	**AMY**	1 ppb	0.0021	**
			10 ppb	< 0.0001	****
			100 ppb	< 0.0001	****
		**AMO**	1 ppb	< 0.0001	****
			10 ppb	< 0.0001	****
			100 ppb	< 0.0001	****
		**SRT**	1 ppb	> 0.9999	n.s.
			10 ppb	0.0376	*
			100 ppb	< 0.0001	****
	Dark Cycle	**AMY**	1 ppb	< 0.0001	****
			10 ppb	< 0.0001	****
			100 ppb	0.08181	n.s.
		**AMO**	1 ppb	< 0.0001	****
			10 ppb	< 0.0001	****

			100 ppb	< 0.0001	****
		**SRT**	1 ppb	0.0967	n.s.
			10 ppb	< 0.0001	****
			100 ppb	< 0.0001	****
Burst Movement Count	Light Cycle	**AMY**	1 ppb	0.2451	n.s.
			10 ppb	< 0.0001	****
			100 ppb	> 0.9999	n.s.
		**AMO**	1 ppb	< 0.0001	****
			10 ppb	< 0.0001	****
			100 ppb	< 0.0001	****
		**SRT**	1 ppb	0.0002	***
			10 ppb	0.0005	***
			100 ppb	< 0.0001	****
	Dark Cycle	**AMY**	1 ppb	< 0.0001	****
			10 ppb	< 0.0001	****
			100 ppb	< 0.0001	****
		**AMO**	1 ppb	< 0.0001	****
			10 ppb	< 0.0001	****
			100 ppb	< 0.0001	****
		**SRT**	1 ppb	< 0.0001	****
			10 ppb	< 0.0001	****
			100 ppb	< 0.0001	****
Rotation Count	Light Cycle	**AMY**	1 ppb	> 0.9999	n.s.
			10 ppb	< 0.0001	****
			100 ppb	< 0.0001	****
		**AMO**	1 ppb	< 0.0001	****
			10 ppb	< 0.0001	****
			100 ppb	< 0.0001	****
		**SRT**	1 ppb	< 0.0001	****
			10 ppb	0.9210	n.s.
			100 ppb	< 0.0001	****
	Dark Cycle	**AMY**	1 ppb	< 0.0001	****
			10 ppb	< 0.0001	****
			100 ppb	< 0.0001	****
		**AMO**	1 ppb	< 0.0001	****
			10 ppb	< 0.0001	****
			100 ppb	< 0.0001	****

		SRT	1 ppb	< 0.0001	****
			10 ppb	< 0.0001	****
			100 ppb	< 0.0001	****

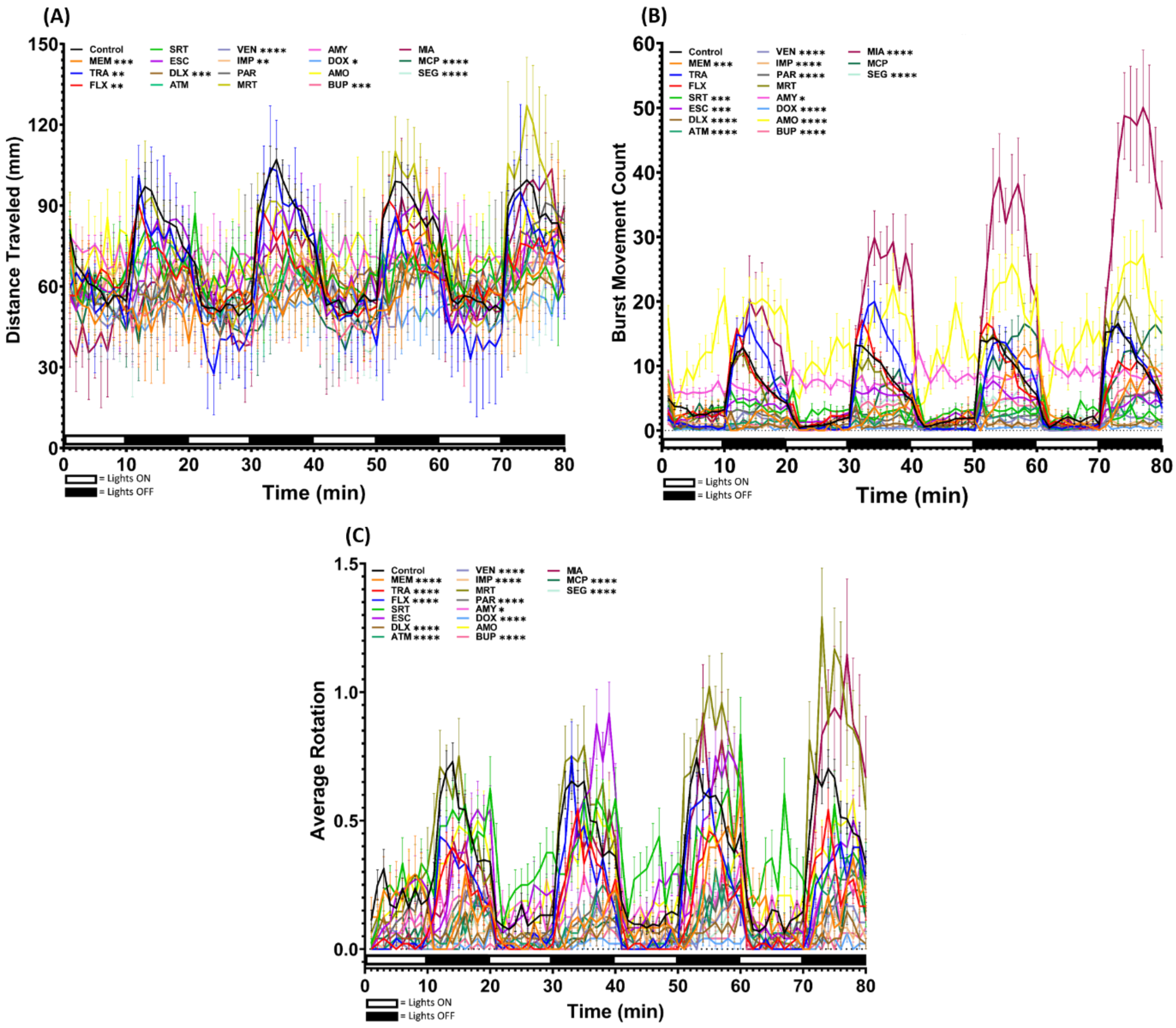

Figure S1. (A) Average distance traveled, (B) average burst movement, and (C) average rotation counts per minute by zebrafish larvae after 1-day exposure of 0 (control) and 1 mg/L of 18 different antidepressants during both light and dark cycles. A two-way ANOVA test was used to compare all treatments with the control. Data are presented as median with 95% CI for (A) and as mean with SEM for (B) and (C) (n = 144 for control, n = 48 for each tested antidepressant; * $P < 0.05$, ** $P<0.01$, *** $P < 0.001$, **** $P < 0.0001$). Abbreviation: Amitriptyline hydrochloride (AMY), amoxapine (AMO), atomoxetine hydrochloride (ATM), bupropion hydrochloride (BUP), doxepin hydrochloride (DOX) duloxetine hydrochloride (DLX), escitalopram oxalate (ESC), fluoxetine hydrochloride (FLX), imipramine hydrochloride (IMP), mianserin hydrochloride (MIA), milnacipran (MCP), moclobemide (MEM), and venlafaxine hydrochloride (VEN), mirtazapine (MRT), Paroxetine (PAR), Selegiline hydrochloride (SEG), Sertraline hydrochloride (SRT), Trazodone hydrochloride (TRA).

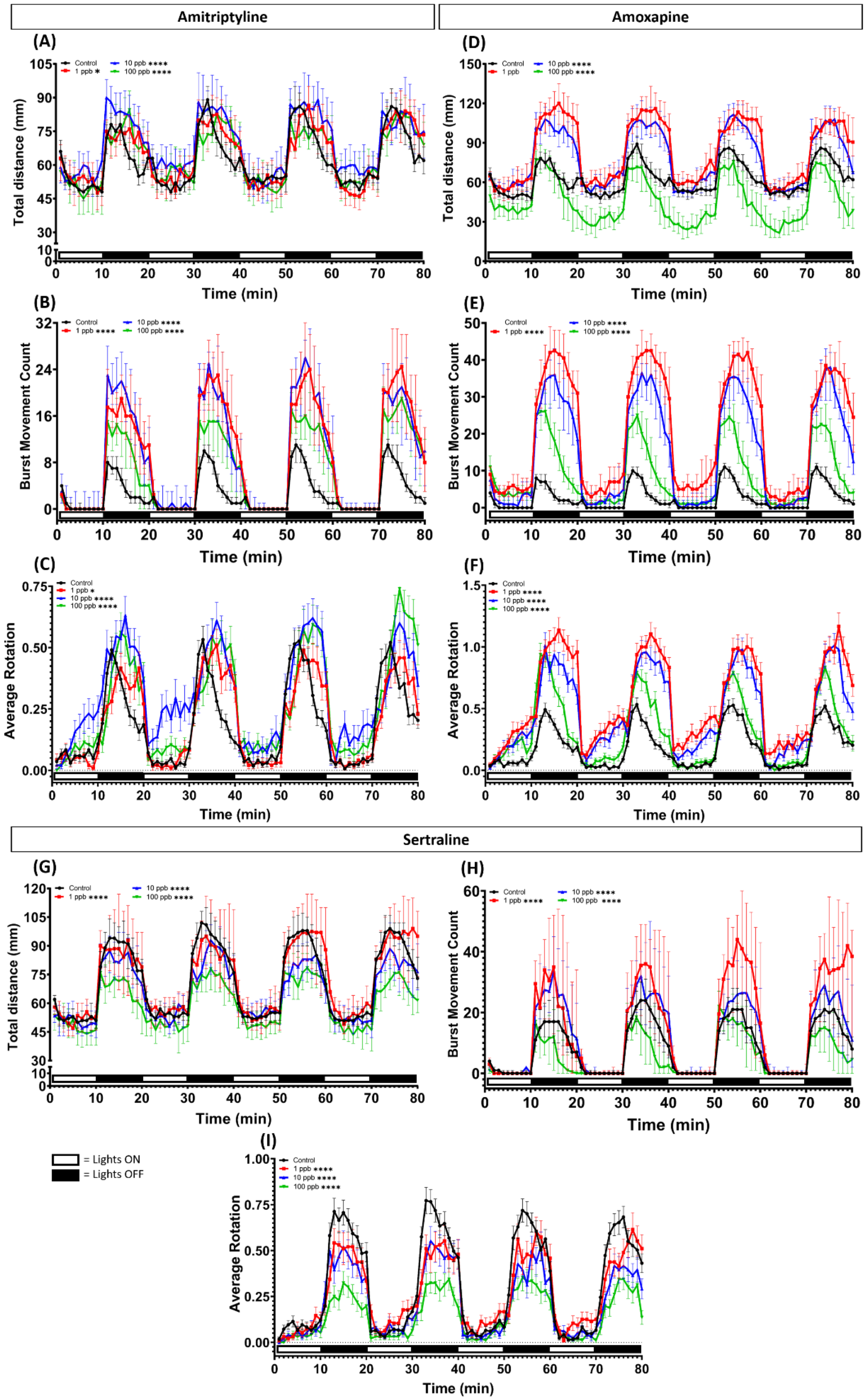

Figure S2. (A, D, G) Average distance traveled, (B, E, H) average burst movement, and (C, F, I) average rotation counts per minute by zebrafish larvae after 1-day exposure of 0 (control), 1 ppb, 10 ppb, and 100 ppb of amitriptyline, amoxapine, and sertraline during both light and dark cycles. A

two-way ANOVA test with Geisser-Greenhouse's correction continued with Dunnett's multiple comparisons test was carried out to compare all treatments with the control. The average distance traveled data are presented as median with 95% CI while the rest of the data are expressed as mean with SEM (n = 167 for control, n = 96 for each concentration of tested antidepressants group, except 10 ppb amitriptyline group (n = 95), * $P < 0.05$, **** $P < 0.0001$).

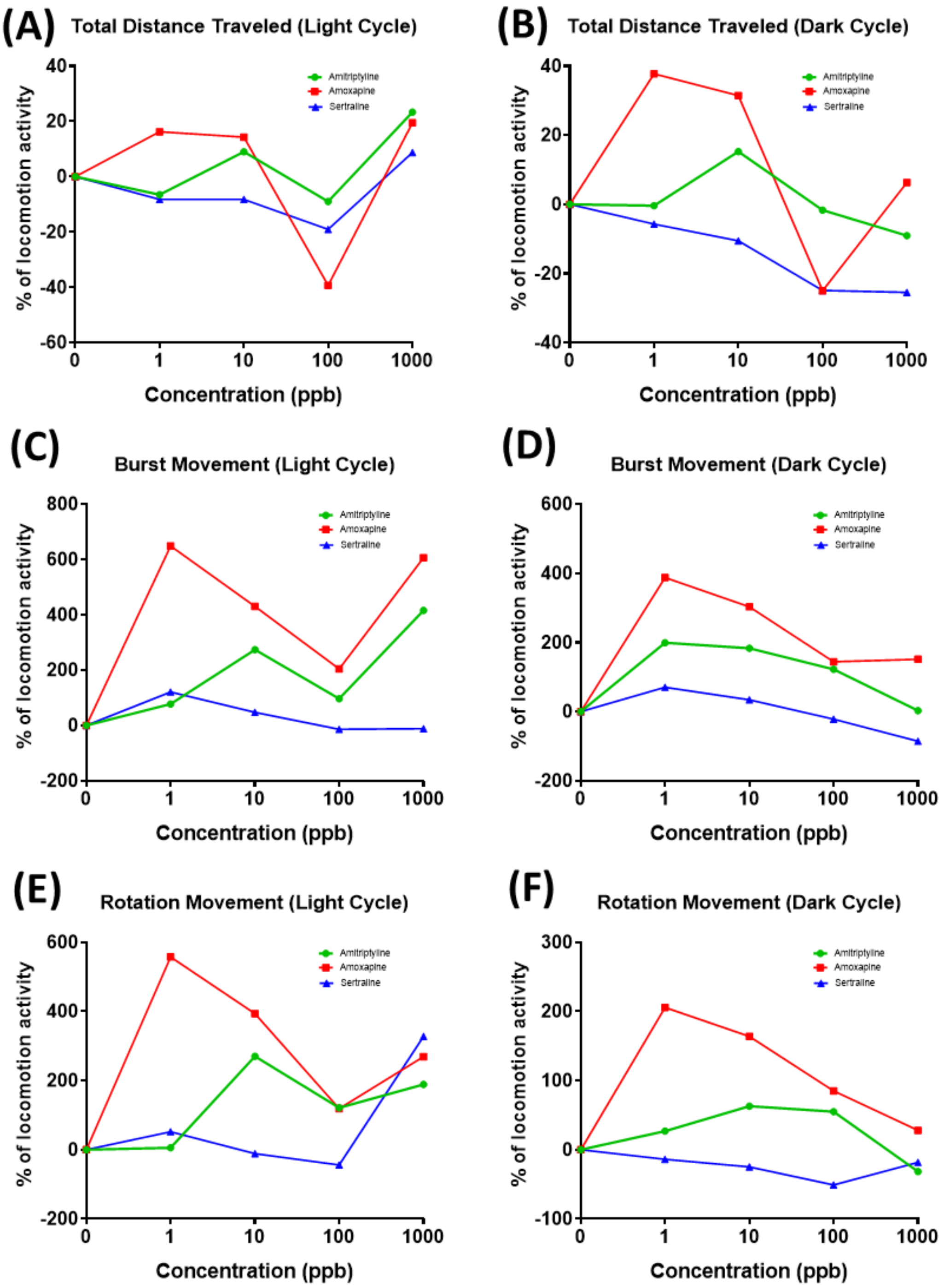

Figure S3. Comparison of non-monotonic dose response curve (A & B) average total distance traveled, (C & D) average burst movement, and (E & F) rotation count of zebrafish larvae after 1-day exposure

of 0 (control), 1 ppb, 10 ppb, and 100 ppb of amitriptyline (green color), amoxapine (red color), and sertraline (blue color) during both light and dark cycles.
